# Wearable Photoplethysmography for Cardiovascular Monitoring: This article summarizes the key literature on wearable photoplethysmography and points to future directions in this field.

**DOI:** 10.1109/JPROC.2022.3149785

**Published:** 2022-01-27

**Authors:** Peter H. Charlton, Panicos A. Kyriacou, Jonathan Mant, Vaidotas Marozas, Phil Chowienczyk, Jordi Alastruey

**Affiliations:** Department of Biomedical EngineeringSchool of Biomedical Engineering and Imaging SciencesKing’s College London, King’s Health Partners London SE1 7EU U.K.; Research Centre for Biomedical Engineering, CityUniversity of London 3163 London EC1V 0HB U.K.; Department of Public Health and Primary CareUniversity of Cambridge 2152 Cambridge CB1 8RN U.K.; Department of Electronics Engineering and the Biomedical Engineering Institute, Kaunas University of Technology 70309 44249 Kaunas Lithuania; Department of Clinical PharmacologyKing’s College London London SE1 7EH U.K.

**Keywords:** Cardiovascular (CV), photoplethysmogram (PPG), pulse wave, sensor, signal processing, smartwatch

## Abstract

Smart wearables provide an opportunity to monitor health in daily life and are emerging as potential tools for detecting cardiovascular disease (CVD). Wearables such as fitness bands and smartwatches routinely monitor the photoplethysmogram signal, an optical measure of the arterial pulse wave that is strongly influenced by the heart and blood vessels. In this survey, we summarize the fundamentals of wearable photoplethysmography and its analysis, identify its potential clinical applications, and outline pressing directions for future research in order to realize its full potential for tackling CVD.

## I. Introduction

Cardiovascular disease (CVD) is a major burden on individuals and societies worldwide. In 2015, there were an estimated 422 million cases of CVD and 18 million deaths due to CVD [1]. Several effective strategies have been identified to reduce cardiovascular (CV) risk, including drugs, such as antihypertensives, lipid-lowering agents, and anticoagulants, and lifestyle changes, such as regular exercise, improved diet, and weight control [2]. Approaches to identify individuals at risk of CVD could prompt these interventions and help reduce CVD-associated mortality and morbidity.

The proliferation of smart wearables equipped with photoplethysmography sensors provides an opportunity to monitor CV health in daily life. Photoplethysmography has already had a profound impact on clinical care through its use in pulse oximeters, which are routinely used to assess blood oxygen saturation in a wide range of clinical settings. The photoplethysmogram (PPG) signal is a measure of arterial blood volume, which fluctuates with each heartbeat and is used by many wearables to monitor heart rate (HR). The PPG also contains information on the cardiac, vascular, respiratory, and autonomic nervous systems. Consequently, signal processing techniques have been developed to extract additional physiological parameters from the PPG. If these techniques could be refined and validated for use in daily life, then potentially wearables could be used for CV monitoring and to inform clinical decisions.

Current trends indicate that, in the future, smart wearables may be even more widely used. The number of people using wearables is growing rapidly [3]: it is estimated that there will be over one billion wearable devices in 2022, rising from 526 million in 2017 [4]. Furthermore, it is predicted that global spending on wearables will exceed $\$ $80 billion in 2021 [5]. Notwithstanding the potential barrier of cost, it is envisaged that wearables could be used for widespread CV monitoring, benefiting both individuals and society.

This survey presents a review of wearable photoplethysmography. It is a narrative review, summarizing key literature on the topic. The review is structured as follows. Section II provides an introduction to photoplethysmography. Section III presents an overview of PPG signal processing. Section IV details potential clinical applications of wearable PPG-based devices. Section V describes directions for future research. Section VI lists resources available to aid researchers in the field. Recommended further reading is provided in Table 1, including key review papers by Allen (written before wearable photoplethysmography devices were available) [6] and Sun and Thakor (which focused on noncontact photoplethysmography) [7].

**TABLE 1:** Further Reading on Photoplethysmography

Topic	Details	Ref
Fundamentals of photoplethysmography		
Photoplethysmography	the technology	[6]–[8]
Signal processing	overview	[9], [10]
Wearable devices	overview	[11]
Physiological measurements		
Heart rate	mathematical techniques	[12], [13]
	performance of devices	[14]
	large-scale study	[15]
Oxygen saturation	overview	[16]
Pulse rate variability	vs. heart rate variability	[17]
Blood pressure	overview	[18]
	pulse wave analysis	[19]
	pulse transit/arrival time	[20]–[22]
	machine learning	[23]
Respiratory rate	overview	[24]
Arterial stiffness	overview	[25]
Vascular age	overview	[26]
Clinical applications		
Applications	overview	[6]
Atrial fibrillation	overview	[27], [28]
	large-scale studies	[29], [30]
Infectious disease	surveillance	[31]
Vascular aging	algorithm development	[32]
Cardiovascular risk	performance assessment	[33]

## II. Photoplethysmography

This section introduces photoplethysmography, a noninvasive, optical technique for measuring the PPG [6].

### A. Photoplethysmography in Clinical Practice

Photoplethysmography was first developed in the 1930s [6]. Its potential applications in CV monitoring were quickly realized, as it was soon proposed that it could be used to identify differences in arterial elasticity between healthy and diseased subjects [34]. It was not until almost half a century later, in the 1980s, that photoplethysmography entered widespread clinical use in the form of pulse oximeters [35]. Pulse oximeters have had a profound impact on clinical care, initially enabling continuous oxygen saturation monitoring during anesthesia, which had “almost never been done before” [36]. They are now used in a wide range of clinical settings [37]. In intensive care, pulse oximeters are often used for HR monitoring in newborn babies [38] and can help avoid exposing premature infants to dangerous levels of supplementary oxygen, which can lead to blindness [39]. In hospital wards, pulse oximeters are used to obtain HR and oxygen saturation measurements in acutely ill patients, which can be used to identify early signs of clinical deterioration [40]. In primary care, they are used to assess respiratory diseases [41]. The role of pulse oximeters continues to grow, as they have recently been recommended for remote management of COVID-19 in the home [42]. There are many more potential clinical applications of photoplethysmography besides pulse oximetry, which are in various stages of development (see Section IV).

### B. PPG Signal

#### 1) Acquisition:

**Fig. 1.**
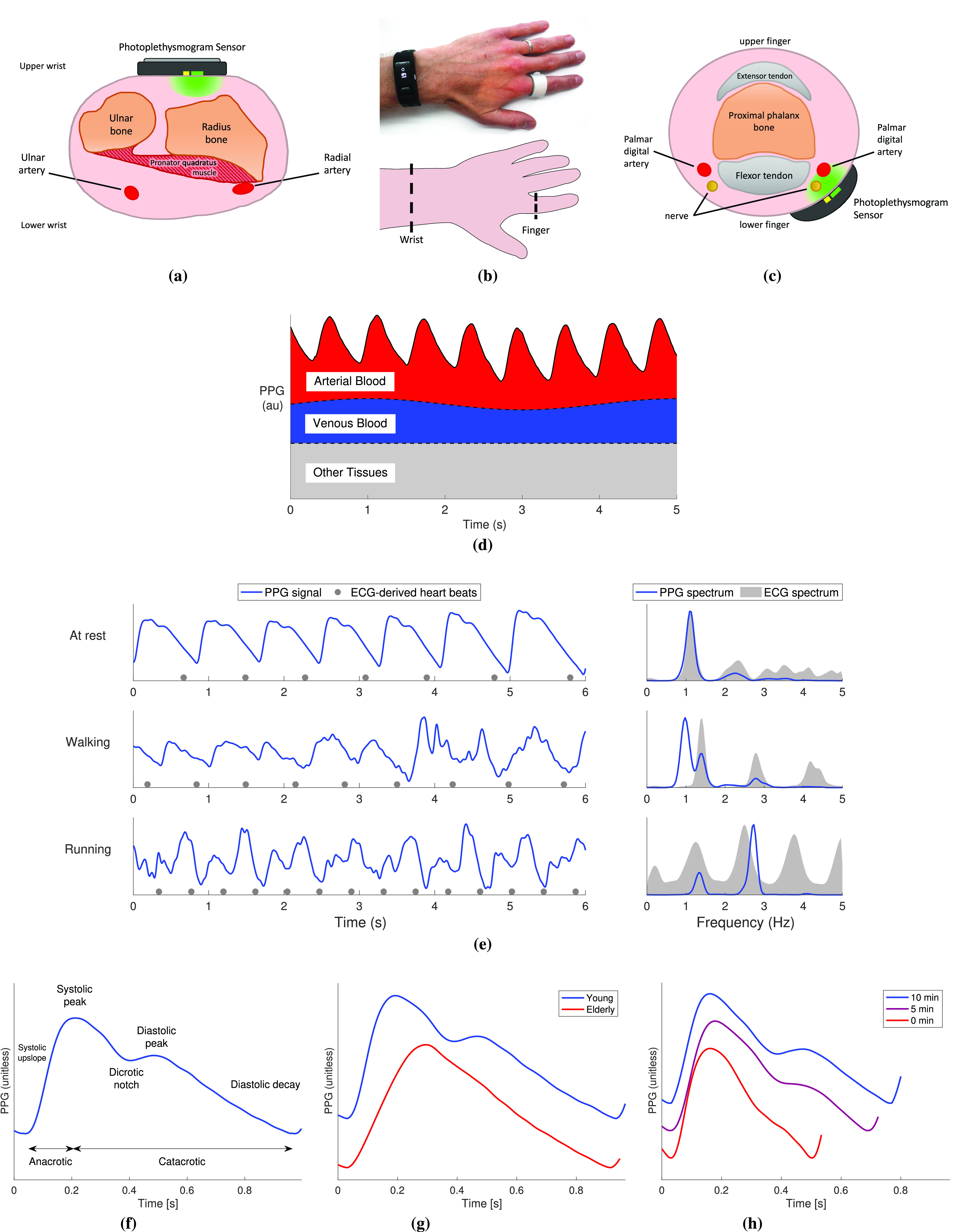
Acquiring PPG signals. (a)–(c) Cross sections of the wrist and finger showing Typical configurations for acquiring PPG signals. (b) Wearable PPG sensors. (d) Physiological Origins of the PPG signal, showing attenuation of light due to pulsatile arterial blood, venous blood, and other tissues. (e) Comparison of PPG recordings (left) and their frequency spectra (right) with electrocardiogram (ECG) measurements during different activities, acquired using infrared reflectance photoplethysmography at the finger. (f)–(h) Key features of a PPG pulse wave and how they change with age and exercise. See the Supplementary Material for additional details and images. Sources: (a) and (c) are adapted from [43] (public domain), (b) is adapted from https://freesvg.org/vector-drawing-of-outline-of-a-raised-hand (public domain), (d) is adapted from [44] under CC BY 4.0, (e)–(h) data from the Vortal dataset, acquired at the finger [45], and (f) is adapted from [46] under CC BY 4.0.

The PPG is a signal comprising information on arterial blood volume. It is obtained by measuring the light either reflected from, or transmitted through, a tissue bed. These two approaches are known, respectively, as reflectance and transmission mode photoplethysmography. Pulse oximeters often use transmission mode, whereas wearables often use reflectance mode. For instance, smartwatches and fitness bands acquire the PPG in the reflectance mode by illuminating the skin at the wrist using an LED and measuring the amount of reflected light using a photodetector [see Fig. 1(a)]. Smart rings use a similar approach at the finger [see Fig. 1(c)]. The PPG can also be measured using a camera, either in contact with the skin (such as by placing a finger on a smartphone camera) or from a distance [7]. The range of inexpensive options for acquiring the PPG signal makes it an attractive technology for health monitoring.

#### 2) Origins:

The PPG signal is produced through the complex interaction of light with multiple tissue components [47]. As shown in Fig. 1(d), it consists of a baseline component and a pulsatile portion. The pulsatile portion of the PPG is primarily determined by the volume of arterial blood in the tissue bed, which increases and decreases with each heartbeat. Fluctuations in venous blood volume also affect the signal.

Other factors that influence the PPG include the temperature [48], [49], the use of reflected or transmitted light, and the anatomical measurement site [50]. The wavelength of light also affects the PPG measurement [18] since green light penetrates less deeply than red and infrared light [51]. Breathing also influences the PPG signal at subcardiac frequencies in three ways (see the Supplementary Material): amplitude modulation, frequency modulation, and baseline wander [24]. The PPG signal is susceptible to motion artifact, as shown in Fig. 1(e), and good skin contact is required to obtain high-quality signals [7]. Peripheral vasoconstriction can also result in low-quality signals [52]. Finally, the PPG can be affected by changes in the height of measurement relative to the heart [53]. These factors should be taken into account when designing sensors to obtain CV measurements.

The shape of PPG pulse waves contains much physiological information. The shape of a PPG pulse wave is determined primarily by the heart and blood vessels. Major features of the pulse wave are shown in Fig. 1(f). Exemplary changes in its shape with age and during recovery from exercise are shown in Fig. 1(g) and (h), respectively. The shape is influenced not only by the incident wave transmitted through the arteries from the heart but also by reflected waves from a range of arterial sites [6]. While there are similarities between PPG and arterial blood pressure (BP) pulse waves, their shapes differ [54] because the signals are produced by different physiological mechanisms. The mechanisms underlying the PPG pulse wave shape are not yet fully understood [6].

#### 3) Measurement Units:

There is no widely accepted measurement unit for the PPG signal. PPG signals are often reported as being unitless or in arbitrary units (as in this article). PPG signals can also be expressed in Volts, as the light intensity detected by PPG sensors is commonly converted to a voltage through a photodiode. In this case, the pulsatile component of the PPG can be of the order of 1 mV in amplitude [55] although lower amplitudes can be caused by technical factors, such as inappropriate gain control, or physiological factors, such as vasoconstriction [9].

It could be beneficial to standardize PPG measurement units to facilitate intersubject and intrasubject comparisons. Potential approaches include, first, expressing the PPG signal as a fraction of the signal obtained without attaching the device to the body. For instance, in the transmission mode, a calibration reading could be obtained without the finger being inserted into the probe and subsequent measurements expressed as a proportion of this initial reading. Second, a “decibels relative to full-scale” approach could be used, expressing the amplitude of the pulsatile component as a proportion of the system’s operating range. Third, amplitudes of PPG features could be normalized by the PPG’s baseline amplitude (i.e., offset)—an approach already used to calculate the perfusion index [56]. Any approach to apply measurement units to the PPG should take into account that the measured light intensity can be affected by factors such as ambient light intensity, transmitted light intensity (which devices can vary dynamically), and sensor contact.

### C. Wearable PPG Device Design

The design of wearable PPG devices must take into account several considerations, such as signal quality, user acceptability, cost, and power consumption. Key design considerations are now summarized, and the reader is referred to [11] for a more detailed treatment.

#### 1) Measurement Site:

The choice of PPG measurement site influences both user acceptability and the usefulness of PPG signals. Many consumer devices are worn on the upper wrist, a site that is widely accepted [57]. However, the PPG measured at the upper wrist differs from that at other sites. First, the pulse wave shape at the upper wrist differs from that at the finger [58], [59]. This may be because the upper wrist PPG is dominated by microvascular blood, whereas the finger PPG is more strongly influenced by blood flow in the digital arteries [see Fig. 1(a) and (c)]. Second, the signal amplitude at the upper wrist is lower than that at other sites, such as the underside of the wrist [55]. Alternative measurement sites include the arm [60] (mounting the sensor in an armband), the outer or inner ear [61], [62] (earbuds), the chest (chestband or adhesive patch), or the face (smart glasses). These alternative sites can have advantages over the wrist, such as being less prone to motion artifact [63] or vasoconstriction [62], and providing more accurate HR measurements [64].

#### 2) Sensor Configuration:

The configuration of a PPG sensor has a large impact on the PPG signal quality. The design should maximize the proportion of light passing through the tissue region of interest while minimizing the influence of ambient light and the amount of light scattered from the skin surface. Key considerations include [55] the geometry of LED and photodiode positioning, since configurations in which LEDs surround the photodiode result in higher PPG signal amplitudes, the spacing between an LED and the photodiode, since a shorter spacing results in higher amplitudes, and the use of an optical barrier between the LED(s) and photodiode, which can increase the signal amplitude.

PPG signal quality can also be improved by acquiring multiple PPG signals from a single sensor. Multiple PPG signals can be obtained by either: 1) having multiple sets of LEDs and photodiodes in different positions or 2) having multiple LEDs surrounding a single photodiode and illuminating each sequentially to obtain PPGs with different measurement paths. A composite PPG signal can then be obtained by combining the individual signals by: 1) averaging with all signals weighted equally [65] or according to their quality [55] or 2) using decomposition to extract significant feature components [66].

There are also benefits to acquiring other signals simultaneously with the PPG, such as the electrocardiogram (ECG) (for pulse arrival time (PAT) measurement), and an accelerometry, gyroscope, or second PPG signal (for motion artifact removal—see Section III-A2).

#### 3) Wavelength of Light:

The wavelength of light emitted by the LED(s) in a PPG sensor has an impact on the resulting signal. Common wavelengths include green (the shortest), red, and infrared (the longest). Longer wavelengths penetrate to deeper depths [47] and are, thus, influenced by different levels of the vasculature. Consequently, red and infrared wavelengths are typically used for transmission mode photoplethysmography. In contrast, green is often used in reflectance mode, resulting in higher quality PPG signals [67], greater robustness to temperature changes [48], and more accurate HR monitoring [68].

#### 4) Sensor Attachment:

The way in which PPG sensors are attached to the body influences the PPG signal. First, it is important to maintain good contact with the skin. Flexible and adhesive sensors are emerging, which may improve contact and, consequently, the signal quality [55], [69]. Second, the contact pressure resulting from the attachment can influence pulse wave shape and timing, meaning that it should ideally be kept constant or calibrated when performing analyses using these signal characteristics [70]. Higher contact pressures have been found to increase the accuracy of HR monitoring during exercise [71].

## III. PPG Signal Processing

This section presents steps to preprocess PPG signals, extract pulse wave features, estimate physiological parameters, and use machine learning to develop PPG signal processing techniques.

### A. Preprocessing

The following preprocessing steps are often taken prior to analysis.

#### 1) Digital Filtering:

**Fig. 2.**
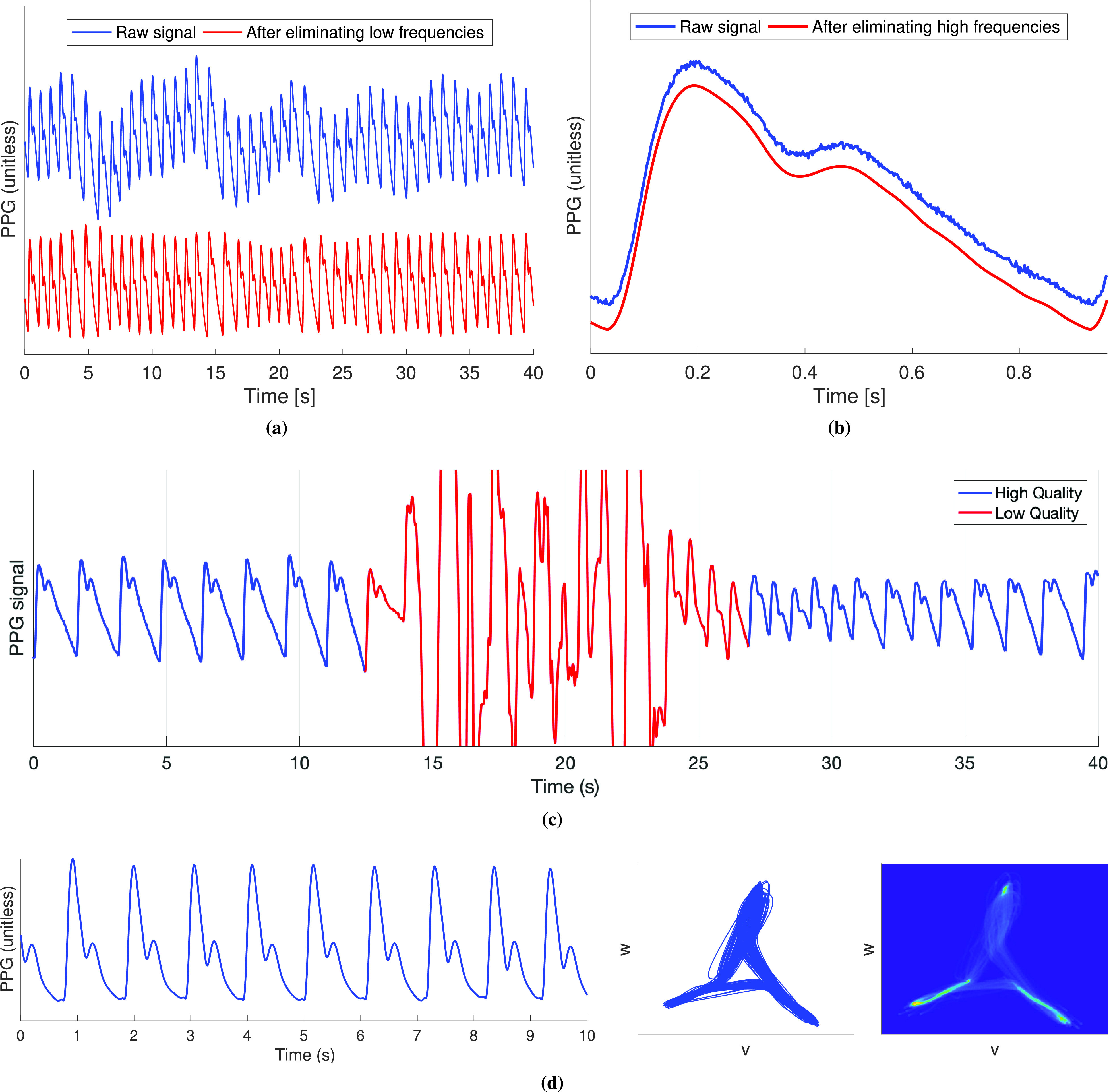
Preprocessing PPG signals. (a) Filtering to eliminate low-frequency content. (b) Filtering to eliminate high-frequency content. (c) Assessing PPG signal quality: segment of PPG signal containing a period of low-quality signal (red) from which pulse wave features cannot be reliably extracted. (d) Representing a PPG signal in phase space using symmetric projection attractor reconstruction. Sources: (a) and (b) data from the Vortal dataset, acquired using infrared reflectance photoplethysmography at the finger [45], (c) data from the PPG diary study [72], and (d) data from the Vortal dataset, acquired at the finger using a clinical monitor [73].

The PPG signal may be filtered to eliminate irrelevant frequency content [74], such as high-frequency noise or low-frequency baseline wander, as shown in Fig. 2(a) and (b). The choice of filter technique and filter order can affect PPG morphology [74]. Therefore, filter cutoff frequencies should be chosen according to the analysis. High-frequency noise can be eliminated using a low-pass filter. A low-pass cutoff frequency as low as 2.25 Hz may be suitable for HR estimation [75], whereas higher cutoff frequencies are required for analyses of pulse wave timing (such as 5 Hz for interbeat interval (IBI) calculation [76]) and pulse wave shape (such as 20 Hz for feature extraction [77]) [11]. Low-frequency baseline wander can be eliminated using a high-pass filter. For HR estimation, the high-pass cutoff should be less than the HR (e.g., 0.4 Hz [75], ensuring that even the lowest plausible HR of 30 bpm is preserved). In contrast, a much lower cutoff frequency is required for respiratory rate (RR) estimation (e.g., 0.05 Hz [24], ensuring that the lowest plausible RR of 4 bpm is preserved). Consequently, different filtering strategies may be required to obtain different parameters (such as HR, BP, and RR).

#### 2) Motion Artifact Removal:

Techniques for removing motion artifacts from the PPG fall into two categories: those which use a single PPG signal and those which use a simultaneous reference motion signal alongside the PPG signal. Motion artifacts can be removed from a single PPG signal using techniques, such as a periodic moving average filter [78], waveform synthesis through stochastic modeling [79], adaptive filtering, and signal decomposition [80]. Reference motion signals that can be used include accelerometry [81], [82], the gyroscope signal [83], or a PPG signal of a different wavelength (such as using an infrared PPG to remove motion artifact from a green PPG) [84]. For further details, see [13] and [85]. Motion artifact removal is particularly helpful for facilitating HR monitoring during exercise [13] although, in many situations, it is preferable to only estimate parameters from high-quality signals to increase accuracy.

#### 3) Signal Quality Assessment:

Signal quality assessment can be used to identify periods in which parameters cannot be reliably estimated, as shown in Fig. 2(c). Several approaches have been proposed, including statistical analysis of pulse wave shape [56], assessing the level of perfusion through the perfusion index (the ratio of the amplitude of the pulsatile component of the PPG to its baseline) [56], assessing the similarity of successive pulse wave shapes using template matching [86] or dynamic time warping [87], and deep learning [88]. For further details of techniques, see [10]. Typically, signal segments are deemed to be either high or low quality through the use of empirical thresholds. It is important to select a threshold suitable for the intended application, as a very high-quality signal might be required for analysis of pulse wave shape, whereas a lower quality signal could be acceptable for HR monitoring.

Wearables already assess PPG signal quality in order to determine whether HR values are accurate [86]. Similarly, novel techniques have been developed to determine whether derived RRs are accurate [89]. Additional techniques will be required to determine which pulse waves can be used to obtain reliable CV parameters.

### B. Identifying Individual Pulse Waves

Individual pulse waves must be identified in order to obtain IBIs (the time between consecutive pulse waves) and extract pulse wave features from individual waves. Approaches to identify individual pulse waves in the PPG seek to overcome two key challenges: 1) pulse waves can exhibit two peaks, particularly in young subjects [see Fig. 1(g)] and 2) artifacts can create spurious peaks [see Fig. 1(e)]. Most approaches start by bandpass filtering the PPG to attenuate noncardiac frequencies. The bandwidth can be determined by the range of plausible HRs (such as 0.4–2.25 Hz [75]) or an initial HR estimate, HR_*i*_ (such as 0.9 ${\text {HR}_{i}}$—2.5 ${\text {HR}_{i}}$ [90]).

The following approaches have been used to identify pulse waves.

#### 1) Detect Maxima or Minima in the PPG:

Maxima (or minima) are detected as markers of candidate pulse waves [75], [91]–[97]. Candidate pulse waves are only accepted as true pulse waves if the maxima (or minima) exceed an adaptive threshold [94], [95] or if the pulse waves have reasonable amplitude and duration [75], [91], [97].

#### 2) Compare Weakly and Strongly Filtered PPGs:

Candidate regions containing pulse wave peaks are identified as time periods of sufficient duration when a weakly filtered PPG (with a higher low-pass cutoff) is above a strongly filtered PPG (with a lower low-pass cutoff). The moving average schemes can be designed according to the typical durations of the systolic peak and heartbeat, respectively [98].

#### 3) Identify Line Segments Indicating Systolic Upslopes:

**Fig. 3.**
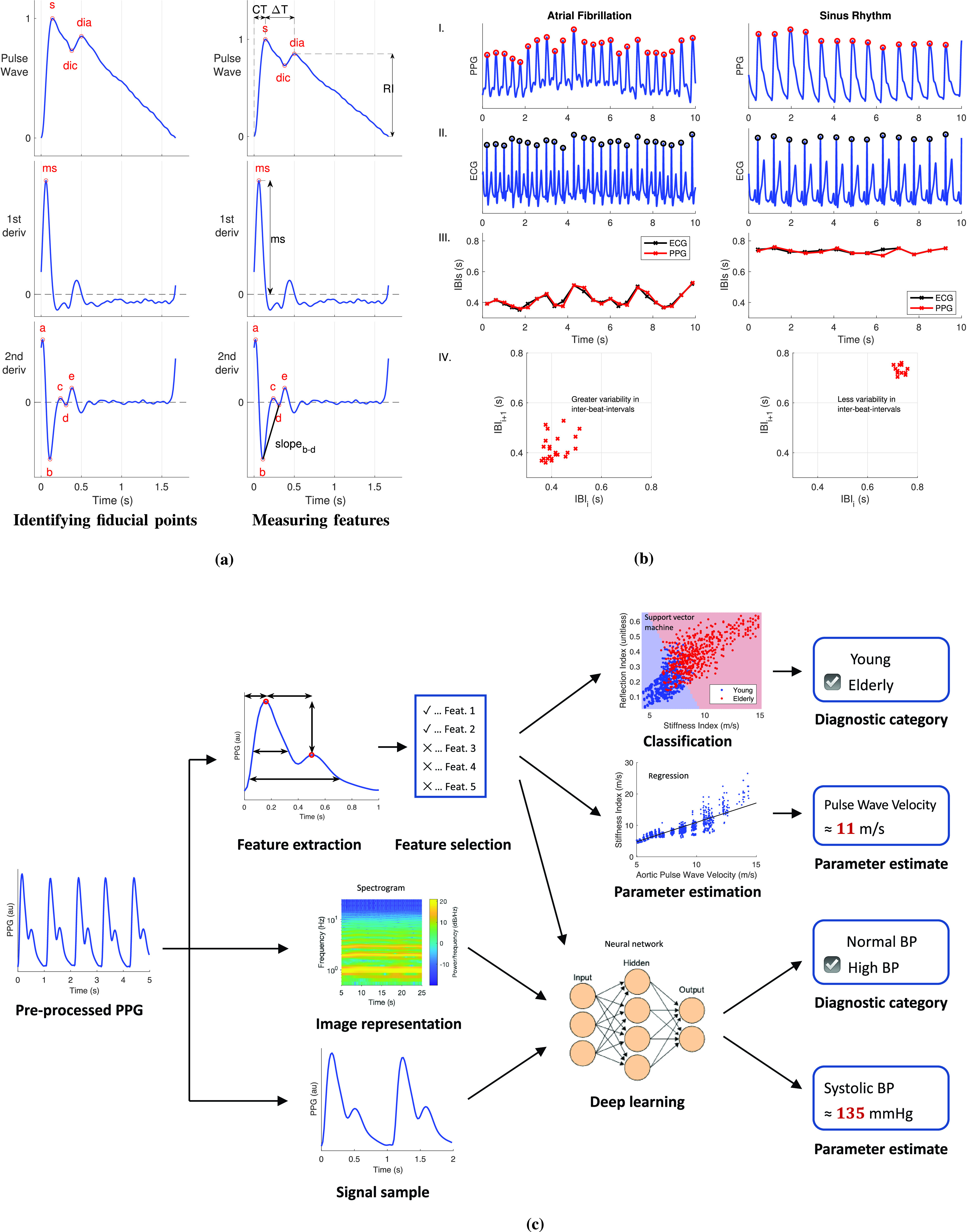
Processing PPG signals. (a) Two steps in extracting features from PPG pulse waves and their derivatives: (left) identifying fiducial points and (right) extracting feature measurements (extracting features from PPG pulse waves). (b) Typical process for detecting AF from PPG signals by quantifying IBI variability using the spread of points on a Poincaré plot (lowest plots, labeled IV.) with ECG signals shown for comparison—these plots all show data from the same subject with the left-hand plots showing data while in AF and the right-hand plots showing data while in normal sinus rhythm. (c) Typical processes for developing models to analyze PPG signals using statistical modeling or machine learning. Sources: (a) is adapted from [113] under CC BY 3.0 (DOI: 10.1088/1361-6579/aabe6a), (b) is adapted from [44] under CC BY 4.0, and (c) is produced using data from the Vortal dataset acquired at the finger using a clinical monitor [73], data from the PWDB Database [114], and C. Burnett’s Artificial neural network diagram (https://commons.wikimedia.org/wiki/File:Artificial_neural_network.svg) under CC BY-SA 3.0.

Candidate systolic upslopes are identified as PPG segments with a continuously positive gradient [99]–[101] [see the systolic upslope in Fig. 3(a)]. Segments must be of sufficient duration and acceptable amplitude to be accepted as true upslopes [99]. The requirement that the gradient is *continuously* positive has been relaxed to increase robustness to noise [102].

#### 4) Detect Maximum Upslopes Using the First Derivative:

Candidate points indicating systolic upslopes are detected from maxima in the first derivative of the PPG [90], [103]–[106] [see the *ms* point in Fig. 3(a)]. Only those above an adaptive threshold are accepted [107], which can be time-varying [95].

#### 5) Identify Systolic Upslopes Using the First Derivative:

Systolic upslopes can be identified from pairs of positive- and negative-gradient zero-crossings in the first derivative, which satisfies adaptive amplitude and duration thresholds [98].

#### 6) Identify Systolic Upslopes Using a Slope Sum Function:

A slope sum function is designed to amplify upslopes and attenuate the remainder of the signal, allowing systolic upslopes to be identified with an adaptive threshold [98].

#### 7) Identify Pulse Onsets Using a Wavelet Transform:

A wavelet transform is used to identify regions containing pulse onsets [108].

#### 8) Analyze the Local Maxima Scalogram:

The local maxima scalogram is analyzed to detect pulse peaks [109], [110].

The reader is referred to [111] for further discussion on approaches for identifying individual pulse waves. It is not yet clear which approaches perform the best nor whether a single approach can be used for all patient groups and recording settings. Some approaches, such as analyzing the local maxima scalogram, use minimal heuristic information, which may make them more suitable for use across settings, although heuristics may be useful for ensuring high performance in particular settings. A comparison of open-source beat detectors on polysomnography data is presented in [112], and work is ongoing to assess their performance across a range of settings, such as during exercise and in the presence of arrhythmias.

### C. Extracting Pulse Wave Features

#### 1) Pulse Wave Analysis:

Pulse wave analysis is commonly used to analyze the PPG. Pulse wave features are extracted in two steps.

First, fiducial points (points of interest) are identified on the pulse wave or its derivatives, as shown in Fig. 3(a). The feet and systolic peak are simply detected as the minima and maximum, whereas reliable detection of the dicrotic notch and diastolic peak requires the use of derivatives [113]. The second derivative is commonly characterized by five fiducial points, named $a$– $e$ [9], [115], [116], which span the time from early systole until the dicrotic notch. Derivatives are typically derived from the PPG after eliminating high-frequency noise, which can otherwise greatly obscure the derivatives. This low-pass filtering is a compromise between retaining the original signal features and increasing robustness to noise [10].

Second, features are measured from timings and amplitudes of fiducial points, as shown in Fig. 3(a). Features measured from amplitudes are often normalized by either the pulse wave amplitude or, for the second derivative, the amplitude of $a$. Once pulse wave features have been extracted, physiological parameters can be estimated from them. It is important to only use high-quality signals for pulse wave analysis, as pulse wave features can be distorted in the presence of noise.

#### 2) Analysis in Phase Space:

An alternative approach is to analyze the PPG in phase space, as illustrated in Fig. 2(d) [117]–[119]. The figure shows a transformation of a PPG signal into phase space using symmetric projection attractor reconstruction. The attractor is defined using the coordinates $v$ and $w$, which are calculated from three points on the PPG signal, each separated by one-third of a heart period [119]. The attractor’s shape is determined by the shape of the pulse waves, and its density is determined by variability in pulse wave shape [117], [118]. Techniques are being developed to extract meaningful features from the attractor, such as by analyzing its size, shape, rotation, and density. It has been proposed that this technique may have advantages over other techniques for quantifying variability in physiological signals, as it uses the entire signal rather than solely IBIs (as is the case in HR variability (HRV) analysis) [120]. Potential applications include the detection of arrhythmias.

### D. Estimating Physiological Parameters

Techniques for estimating key physiological parameters from the PPG are now described.

#### 1) Heart Rate:

HR is already widely measured by wearable PPG devices. The HR is measured as the pulse rate (PR)—the rate of pulse waves detected in the PPG. The PR is commonly estimated by denoising the signal to reduce motion artifact, identifying its fundamental frequency [which corresponds to HR in high-quality PPG signals, such as those acquired at rest in Fig. 1(e)] and using a tracking algorithm to ensure that PR estimates do not differ greatly from one window to the next [12]. PR can also be calculated from IBIs (e.g., the time between systolic peaks).

The performance of several wrist-worn devices for estimating HR is reviewed in [14] and [121]. HRs have been found to be acceptably accurate during rest [e.g., summary mean absolute error (MAE) of 2.15 beats per minute (bpm)] although they are less accurate during exercise (e.g., MAE of 7.70 bpm during treadmill exercise and 10.64 bpm during cycling) [14]. Furthermore, different devices have different levels of accuracy [122]. Work is ongoing to improve HR estimation techniques in the presence of motion [80] and contextualize HR measurements according to the individual and time of day [15], [123]. For instance, resting HR (HR during periods of inactivity) and sleeping HR have been proposed as useful markers of health [15], [31]. Future studies on the validity of wearable HR devices will benefit from recent guidelines for assessing and reporting performance [124].

#### 2) Pulse Rate Variability (PRV):

PRV is the variability of IBIs derived from a pulse wave signal. PRV and HRV (the ECG-derived gold standard) can each be assessed by: 1) identifying individual heartbeats in the signals (see Section III-B); 2) calculating IBIs; 3) eliminating outlying IBIs (using an approach such as that proposed in [125], which has been applied to the PPG in [126]); and 4) quantifying variability using a range of statistics [127].

Much research has investigated whether PRV can be used as a surrogate for HRV [17], [128]. There is a fundamental difference between HRV and PRV: HRV is obtained from the timings of electrical impulses causing ventricular contraction, whereas PRV is obtained from the timings of pulse waves arriving at the periphery. The time difference between the electrical impulses and pulse wave arrival consists of the preejection period (the time between the impulses and ejection of blood into the aorta) and the pulse transit time (PPT) (the time taken for the pulse wave to propagate from the heart to the measurement site). Both of these components are variable, potentially causing differences between HRV and PRV. Nonetheless, reviews have concluded that PRV is a good surrogate of HRV for certain applications at rest. However, it cannot yet be considered to be a surrogate during exercise or stress [17], [129]. Potential explanations for this include [17]: 1) motion artifact in the PPG rendering PPG-derived IBIs less accurate; 2) a lack of motion artifact removal in PRV studies; and 3) variability in preejection period and PPT causing additional variability in PPG-derived IBIs.

There are several design considerations for PRV assessment. First, the PPG sampling rate should be sufficiently high (at least 25 Hz [130]). Second, the PPG can be interpolated to increase performance [130], [131]. Third, the choice of the fiducial point from which to obtain IBIs impacts performance with the tangent intersection point often giving the best performance [132]–[134].

#### 3) Arterial Blood Oxygen Saturation (SpO_2_):

SpO_2_ can be measured from two PPG signals acquired using different wavelengths of light ( $\lambda _{1}$ and $\lambda _{2}$, which are typically red and infrared) using the expression [135]

\begin{equation*} \text{Modulation ratio},\quad R = \frac {(AC/DC)_{\lambda _{1}}}{(AC/DC)_{\lambda _{2}}}\tag{1}\end{equation*}

where AC and DC are the amplitudes of the pulsatile and baseline components of the PPG signal, respectively, and $R$ is related to SpO_2_ through an empirically determined calibration curve [136]. This approach is widely used in clinical practice to measure SpO_2_ using pulse oximeters. Performance can be improved by filtering the PPG to accentuate the heart frequency [137]. An alternative approach proposed in [138] has been found to provide greater accuracy when using low-quality PPG signals, although at greater computational cost [137].

SpO_2_ measurement is now being incorporated into smart wearables [139], requiring two PPG signals at different wavelengths. There are several challenges to using reflectance mode photoplethysmography to assess SpO_2_: 1) it is difficult to estimate SpO_2_ during motion artifact [140]; 2) it may be difficult to obtain PPG signals of sufficiently large amplitude [141]; and 3) changes in position, contact pressure, and venous blood flow can result in inaccuracies [142]. More generally, SpO_2_ measurements provided by conventional pulse oximeters are susceptible to errors [143], and they have been found to be less accurate and less reliable in black patients than white patients [144]–[147], highlighting the need to improve the technology.

#### 4) Respiratory Rate:

RR, the number of breaths taken per minute, can be estimated from subtle respiratory modulations in the PPG [24]. The respiratory modulation of the PPG can be modeled using three modulations (see the Supplementary Material): baseline wanders (changes in the offset), amplitude modulation (changes in pulse wave amplitude), and frequency modulation (changes in IBIs). Algorithms to estimate RR generally consist of three steps [45]. First, respiratory signals are extracted. These are signals dominated by respiration and are often obtained by measuring the baseline, amplitude, or frequency of each pulse wave. Second, RR is estimated from each respiratory signal using either a time-domain technique (such as detecting individual peaks in the signal) or a frequency-domain technique (such as identifying the fundamental frequency of the signal). Third, multiple RRs can be fused to produce a final estimate. Since respiration has a much smaller influence on the PPG than the heart, it is more difficult to obtain reliable RR estimates than HR estimates. A key challenge is to make algorithms robust to signal artifact and only output values associated with a high degree of confidence [89], [148], particularly when used with wearable data. Recent research has investigated estimating other respiratory parameters from the PPG, such as inspiratory time [149], with potential applications in identifying breathing disorders.

#### 5) Blood Pressure:

BP has been estimated from the PPG using the following inputs [26]: a single PPG [19]; a proximal and a distal PPG to measure PPT [21], [22]; or a distal PPG signal and a signal indicating the time of ventricular contraction (e.g., ECG, phonocardiogram) to measure PAT [20], [21], [150]. Techniques that use a single PPG signal are based on analysis of pulse wave shape using either extracted features [151]–[153] or the whole pulse wave [154]. Often machine learning is used to create a model relating pulse wave features to BP [23]. Techniques that use PTT or PAT require a second signal, such as ECG at the wrist [155] or a signal at the chest. Several models relating BP to PTT or PAT have been proposed [156], including models that incorporate additional variables, such as HR [157]. Models that require a single calibration cuff measurement are convenient but potentially less accurate than those that use multiple measurements to form a patient-specific calibration curve [20]. While frequent calibration may be necessary to accurately estimate absolute BP values from a single PPG, less frequent calibration may be required when estimating BP from certain PAT measurements [158]. Furthermore, it may be possible to identify changes in BP from a single PPG without calibration [159], which could have utility in detecting clinical deteriorations, such as sepsis.

The volume-clamp method is an alternative PPG-based approach for measuring BP [160], [161]. This method consists of: 1) applying an inflatable cuff around the finger; 2) using a PPG sensor to monitor blood volume in the finger; and 3) continuously adjusting the cuff pressure to maintain a constant blood volume. The cuff pressure can then be assumed to be equal to arterial BP. This approach is used in several clinical monitors [162], but it is less suitable for use in wearable devices due to the need for a cuff [19].

Wearables that use the PPG for BP monitoring are widely available. Most devices are not validated [163] although some have recently been certified for medical use [164]–[166]. Studies are now assessing the accuracy and potential clinical utility of such devices [167]. Standards have been developed for the validation of wearable, cuffless BP devices [168], [169] although further work is required to refine validation standards to account for the issues presented by PPG-based devices [170].

#### 6) Arterial Stiffness:

Arterial stiffness is an independent predictor of CV morbidity and mortality [171]. Since it is related to BP, it can be assessed using similar techniques to those used to estimate BP. Most techniques use pulse wave features. Over 30 features have been proposed for assessing the stiffness of both the large and small arteries from a single PPG, including those in Fig. 3(a) [113], [172]. In addition, deep learning has recently been used to develop a model for assessing arterial stiffness from the PPG [173]. If arterial stiffness could be assessed from smart wearables, then, potentially, this approach could be used to monitor vascular age, the biological age of the arteries.

#### 7) Left Ventricular Ejection Time (LVET):

LVET is the duration of systole, the time for which blood is ejected into the systemic circulation in a single heartbeat. It has been proposed that this could be assessed from the PPG pulse wave either from the time between pulse onset and the dicrotic notch ( $e$) [174] or from the PPG’s first derivative [175]. LVET could be useful for monitoring CVDs, such as aortic valve disease and left ventricular failure.

#### 8) Additional Parameters:

Additional physiological parameters have been estimated from the PPG although these techniques are relatively novel. Blood glucose level, widely used for diabetes self-management, has been estimated from PPG pulse wave shape [152], [176], and the pulse wave has been used to classify patients as diabetic or not [177]. Cardiac output, monitored in peri-operative and critical care settings, has been estimated from pulse wave shape and low-frequency PPG variations [6], [178]. Fitness parameters, such as energy expenditure and maximal oxygen consumption ( $\dot{\text{V}}\text{O}_{2\mathrm {max}}$), can be estimated from PPG-derived HRs in combination with other wearable data [11]. The PPG has also been used to assess perfusion [179], [180], hemoglobin concentration [181], and haemorheology [182]. Further research will investigate whether these can be reliably estimated from wearable PPG signals.

### E. Developing Models to Analyze PPG Signals

Statistical modeling and machine learning can be used to develop models to analyze PPG signals [23]. Typical processes for developing models are illustrated in Fig. 3(c). Models either estimate a parameter (such as BP) or provide a diagnostic classification (such as high or normal BP). Machine learning and statistical models typically take pulse wave features as inputs. Deep learning models can also take a signal segment as an input either in its original form or represented as an image. Convolutional and long short-term memory neural networks are often used as deep learning models although it is not yet clear what the best neural network architectures are with which to analyze PPG pulse waves. Deep learning approaches have the following advantages over traditional feature extraction approaches: 1) they are hypothesis-free, i.e., there is no need to hypothesize the pulse wave features of interest and 2) they do not require pulse wave analysis algorithms, which can be difficult to design for good performance across a wide range of conditions (such as different patient states and different PPG acquisition methods). In the future, it will be valuable to assess whether any particular modeling approach provides better performance in a range of clinical applications, and how the performance of such approaches compares to traditional pulse wave analysis techniques.

## IV. Clinical Applications

This section introduces those clinical applications of wearable PPG, which have received recent attention in the literature. Additional applications are described in [11]. Most applications consist of identifying a pathology or obtaining a physiological measurement that can contribute to CV disease diagnosis or prognosis. The potential utility of wearable PPG in these applications lies in its convenience: existing clinical tests can be invasive and more time-consuming, are conducted at a specific time rather than continuously in daily life, and often require a clinical operator and more expensive equipment. In contrast, PPG-based approaches could potentially be performed remotely without direct patient contact and with minimal patient training. However, they usually cannot be considered as a replacement for clinical tests often due to their inferior performance. Therefore, the potential role of PPG-based approaches is likely to be in the early detection of CVD and ubiquitous measurement of risk factors, which could trigger a further clinical assessment.

### A. Detecting Atrial Fibrillation

One of the most promising uses of smart wearables is to detect atrial fibrillation (AF), which can be identified from pulse wave signals due to the irregular heart rhythm produced by AF [27]. AF is one of the most common arrhythmias, diagnosed in 3% of U.K. people over the age of 35 [183]. It is associated with 25%–33% of strokes [184]–[187], and with increased stroke fatality and recurrence rates [186]. It is important to identify AF since untreated AF is associated with up to a fivefold increase in stroke risk [188], [189]. However, it can be difficult to identify AF as it may not produce symptoms and may occur only intermittently [190]. Consequently, it is often not recognized in clinical practice [191], where gold-standard ECG assessments may not be indicated due to a lack of symptoms, and intermittent episodes of AF may be missed as 12-lead ECG assessments are usually conducted at a single time point in a clinic, rather than over several days during daily life.

In AF, compared to sinus rhythm, the PPG signal exhibits greater variability in both IBIs and the shape of the pulse wave, as shown in Fig. 3(b). Several techniques have been proposed to assess the irregularity of IBIs and the shape of pulse waves in order to detect AF from the PPG [27], [28]. In addition, neural networks have been used to detect AF from PPG signals and PPG-derived HRs [192]. The performance of techniques for detecting AF from the PPG has been assessed in several studies [76], [193]–[196] although further work is required to determine which approach is most suitable for clinical use [28].

The potential utility of wrist-worn PPG devices for detecting possible AF has been assessed in recent studies [197], [198]. In a community study [29], a smartwatch was used to identify possible AF in 1-min PPG recordings by extracting IBIs, determining the irregularity of IBIs using a Poincaré plot [see Fig. 3(b) IV.], and generating a notification of possible AF if five out of six consecutive recordings were classified as irregular. The positive predictive value (PPV) of notifications was found to be 0.84, indicating that wearables may have utility for identifying possible AF. Other aspects of the algorithm’s performance (such as sensitivity and specificity) were not assessed. Another study also found a high PPV for possible AF episodes identified from 45- to 60-s PPG recordings [30], [199], and further studies are ongoing [200]. Further work is required to assess the effectiveness and clinical utility of these approaches for opportunistic detection of AF and for AF screening, as discussed in Sections V-D and V-E. Indeed, studies are ongoing to assess the performance of such approaches in a target setting and population [201]. It is most likely that, when used in clinical practice, PPG-based devices could prompt ECG-based assessment when possible AF is detected, rather than being used for decision making on their own [202].

### B. Identifying Obstructive Sleep Apnea

Wearables may also be useful for detecting obstructive sleep apnea (OSA)—the repetitive cessation of breathing during sleep that can occur due to airway collapse. It is estimated that almost one billion people worldwide are affected by OSA [203]. Untreated OSA is associated with an increased risk of stroke, hypertension, and heart failure (HF), among others [204]. However, it is thought that the majority of patients with OSA are undiagnosed due, in part, to the need for overnight tests in a sleep laboratory for definitive diagnosis [205].

Three broad approaches have been proposed to identify apnea events from the PPG. First, desaturations can be detected from SpO_2_ values [206]; a desaturation of 4% is often used as an (imperfect) indicator of apnea [207]. Second, reductions or cessations in breathing can be detected from changes in PPG-derived respiratory modulations [208], which can be obtained from several features of the PPG, including pulse amplitudes, areas, and intervals [209], [210]. Third, the apnea–hypopnea index that is commonly used to diagnose OSA can be estimated from the PPG [211]. The introduction of SpO_2_ monitoring in wrist-worn wearables may make it feasible to perform preliminary overnight OSA tests at scale [206]. In addition, techniques to assess respiratory activity, a marker of disordered sleep, from wearable PPG may be useful for identifying OSA [212].

Evidence is emerging demonstrating the potential utility of wearables for OSA screening. First, a model has been developed to identify OSA using data, which could be obtained from a wearable user (pulse oximetry biomarkers and demographic information) [206]. Second, the performance of this model was found not to be impaired when the reference sleep stages provided by screening in clinical practice were unavailable [213]. A key step now toward using wearables to screen for OSA is to ensure that SpO_2_ monitoring in wearables is accurate (see Section III-D3).

### C. Monitoring the Spread of Infectious Diseases

Infectious diseases, such as influenza, have profound impacts on population health [214]. At the time of writing, the coronavirus (COVID-19) disease pandemic is accounting for widespread mortality, economic damage, and restrictions to daily life. Infectious disease surveillance data can inform policy on issues such as healthcare resource allocation and measures to control the spread of disease. Traditional surveillance systems typically rely on data acquired in healthcare settings, such as clinical diagnoses, laboratory tests, health system usage, and mortality records. These data have the advantages of being of high quality and including specific clinical endpoints. However, they are subject to delays incurred by the reporting processes and the time taken for an infection to result in measured events, such as hospital admission or death.

It has been proposed that wearables could be used for real-time surveillance of infectious diseases, enabling earlier response to disease trends. A recent study demonstrated that influenza surveillance can be improved by incorporating resting HR and sleep durations measured by wearable PPG-based devices into prediction models [31]. Studies have demonstrated the potential utility of smartwatch data for improving detection of COVID-19 beyond that provided by symptom data alone [215] and for presymptomatic detection of COVID-19 [216]. In the future, wearable data could be integrated into surveillance models, gaining all the advantages of the different data sources, to inform population-level decisions [217]. The inclusion of a wide set of variables, including HR, step count, and temperature, as well as novel parameters, such as SpO_2_, RR, and BP, may enhance performance (with SpO_2_ being particularly helpful for detecting hypoxia associated with COVID-19 [42]). If disease detection is accurate enough, then wearable data could also inform individual-level decisions, such as prompting individuals to self-isolate or be tested. In addition, the ability to measure HR, RR, and temperature from a wrist-worn wearable may aid remote diagnosis of community-acquired pneumonia due to COVID-19 [218].

### D. Sleep Monitoring

It has been proposed that smart wearables could be used for sleep monitoring. It is estimated that approximately one-third of adults suffer from sleep disturbance [219]. Insufficient sleep is associated with the development of CV risk factors (such as obesity) and CVD (such as hypertension and heart disease) [220]. Sleep disorders are currently diagnosed through polysomnography, a laboratory-based sleep study involving multiple sensors placed at several points on the body. However, polysomnography is expensive and time-consuming, and uses several, potentially uncomfortable sensors. An alternative is to use a smart wearable in daily life, which could provide earlier detection of disorders and reduce the need for polysomnography.

Several studies have proposed algorithms for classifying sleep stages from the PPG. Algorithms have used PPG-derived HR [221], PRV [222], [223], RR [222], pulse wave morphology [223], and accelerometry in combination with PPG [222]. If sleep stage classification algorithms performed sufficiently well, then smart wearables could identify abnormal sleep patterns and prompt screening for sleep disorders.

### E. Assessing Mental Stress

Mental stress is associated with the development of CVD, and CV morbidity and mortality [113], [224]. Long-term social isolation and job strain are associated with an increased risk of coronary heart disease [225]. Short-term stressors, such as natural disasters and emergency duties, are associated with an increased risk of cardiac death [224], [226]. Stressors are also associated with elevated BP [227], [228] and stroke [189]. However, it is difficult to measure stress frequently in everyday life: salivary cortisol swab tests are often used as a reference marker of stress [229]. This provides an incentive for using wearables to monitor stress and assist with stress management.

Mental stress can be assessed from the PPG using PRV and pulse wave shape features. The utility of PRV for assessing stress has been investigated in several studies [230]–[232], including a study of PRV metrics derived from short recordings [233]. This approach is closely related to that used to assess stress from ECG-derived HRV metrics [234]. In addition, the utility of other pulse wave features has been investigated [230]. While it has been observed that stress tends to increase HR and increase PRV [230], further research is required to determine how reliably PPG features can be used to monitor stress in daily life.

### F. Assessing Vascular Age

The mechanical properties of the aorta change with age: both stiffness and diameter increase with age. In the clinical setting, these changes can be assessed from aortic pulse wave velocity (PWV), the speed of pulse wave propagation along the aorta, since PWV is influenced by both arterial stiffness and diameter. Vascular age can be defined as the apparent age of an individual’s arteries, relative to the age of a healthy subject whose arteries have the same mechanical properties as the individual in question. Vascular age may have particular utility for CV risk prediction since aortic PWV has been found to be predictive of CV events and all-cause mortality [171].

It has been proposed that the PPG could be used to assess vascular age [26], [32]. Several aging indices have been proposed based on features of the second derivative [see Fig. 3(a)]: $d/a$ [235], $(b-c-d-e)/a$ [236], $(b-e)/a$ [236], and $(c+d)/b$ [237]. It is also hypothesized that the stiffness and reflection indices (calculated from the timing and amplitude of the systolic and diastolic peaks) are indicative of vascular aging [235]. Some of these indices have been found to be independent predictors of CV risk and mortality [33], [238], demonstrating their potential utility for risk prediction. If, in the future, wearable data can be automatically entered into an electronic health record, then, potentially, it could be used to augment existing risk predictions and identify patients who may benefit from more detailed CV assessments.

### G. Identifying Clinical Deteriorations

Smart wearables could also be used to identify deteriorations in chronic and acute illnesses since several parameters that are indicative of different organ systems can be derived from the PPG. For instance, HR, RR, and SpO_2_ have previously been derived from the PPG to identify deteriorations in health [239], including changes prior to cardiac arrests [46]. If similar approaches could be implemented in smart wearables, then they could provide early warning of deterioration, facilitating earlier intervention. PPG-based wearables can also facilitate remote physiological monitoring in the acute care setting, reducing the staff contact required to monitor patients with infectious diseases, such as COVID-19 [240].

### H. Cardiovascular Risk Prediction

Smart wearables may also enable the identification of individuals at risk of CVD. The stiffness index (SI) and the $d/a$ ratio calculated from the PPG’s second derivative may be predictive of CV mortality [33], [241]–[243]. If indices were measured by smart wearables, then they could facilitate the early identification of at-risk individuals. This is in contrast to current practice, where CVD screening mostly requires direct contact with patients.

### I. Assessing Response to Exercise

The rate of HR recovery (HRR) after exercise has been associated with CV events and mortality [244], and postoperative morbidity [245]. A slower HRR rate is associated with increased CV risk. Currently, HRR is only routinely measured in exercise stress tests. It may be possible to obtain similar measurements using smart wearables [246] in everyday activities, such as stair climbing [247], or after intense exercise [248].

### J. Identifying Sepsis

Sepsis plays an important role in CVD, contributing to approximately 24% of HF deaths [249], and increasing the risk of CVD for years after infection [250]. Early recognition of sepsis is important for timely administration of antimicrobial therapy, particularly since approximately 70% of inpatients with sepsis acquire the infection in the community [250]. Wearables may provide the opportunity to recognize sepsis earlier by identifying changes in RR, BP, HRV, and temperature, which can occur during the onset of sepsis [251]–[253]. Outstanding research questions include: 1) which markers change consistently during the onset of sepsis; 2) what criteria should be used to identify patients who should be assessed for possible sepsis; and 3) is this approach effective for identifying sepsis?

### K. Identifying Heart Failure

HF is a major cause of CV mortality [254]. Approximately 900 000 people suffer from HF in the U.K., around 40% of whom will die within one year of diagnosis [255]. Early detection of the cardiac dysfunction leading to incident HF may be beneficial since several causes of the dysfunction can be halted or reversed [256]. Alterations in the heart’s response to ventricular preload in HF (the Frank–Starling mechanism) result in measurable differences in PPG wave morphology, particularly in response to standing [175].

If wearables are to be used to identify HF, then it is important to establish thresholds for identifying patients at risk of incident HF from PPG-derived metrics. Several metrics indicative of HF can be calculated from the PPG. First, the speed of HRR in response to standing is reduced in HF and is predictive of mortality [257], [258]. Second, LVET is an independent predictor of incident HF [259]. Furthermore, the reduction in LVET after standing is smaller in HF patients [260]. Third, the increase in cardiac contractility after a prolonged beat (due to an extrasystolic beat or AF) is greater in HF patients [261]. Outstanding research questions include: 1) which metrics are predictive of incident HF and 2) what thresholds should be used to identify patients who warrant screening for HF?

### L. Identifying Preeclampsia

Preeclampsia is a complication occurring in 3%-5% of pregnancies, which is characterized by high BP [262]. It is important to detect it early, as it can result in maternal and fetal morbidity and mortality. The PPG pulse wave shape has been found to differ between pregnant women with preeclampsia and those without [263]. Consequently, it has been proposed that the PPG could be used to identify possible preeclampsia, particularly in resource-constrained settings [264]. Further work is required to develop methods to detect preeclampsia from the PPG and to determine whether they are a useful adjunct to current clinical practice.

## V. Research Directions

We now describe directions for future research to realize the potential of wearable PPG for CV monitoring.

### A. Understanding the Determinants of the PPG Pulse Wave

Research into techniques to assess CV state from the PPG would be greatly aided by a fuller understanding of how CV properties influence pulse wave shape. Ideally, the influence of each property on the PPG would be studied independently. This could be attempted in experiments involving drug administration or physiological maneuvers although it is difficult to ensure that only a single property changes at once in such studies. A complementary approach is to simulate the PPG using modeling. Novel insights obtained using this approach are now presented.

#### 1) Methods:

A computational model was used to simulate the PPG for healthy subjects aged 25–75 under a range of CV conditions [114]. Two pulse wave indices that have been found to be indicative of CV risk were extracted from the pulse wave: the SI and the $d/a$ index. The SI is calculated from the time between systolic and diastolic peaks ( $\Delta T$), and $d/a$ is the ratio of the amplitudes of $d$ and $a$ points on the second derivative, as shown in Fig. 3(a).

#### 2) Results:

**Fig. 4.**
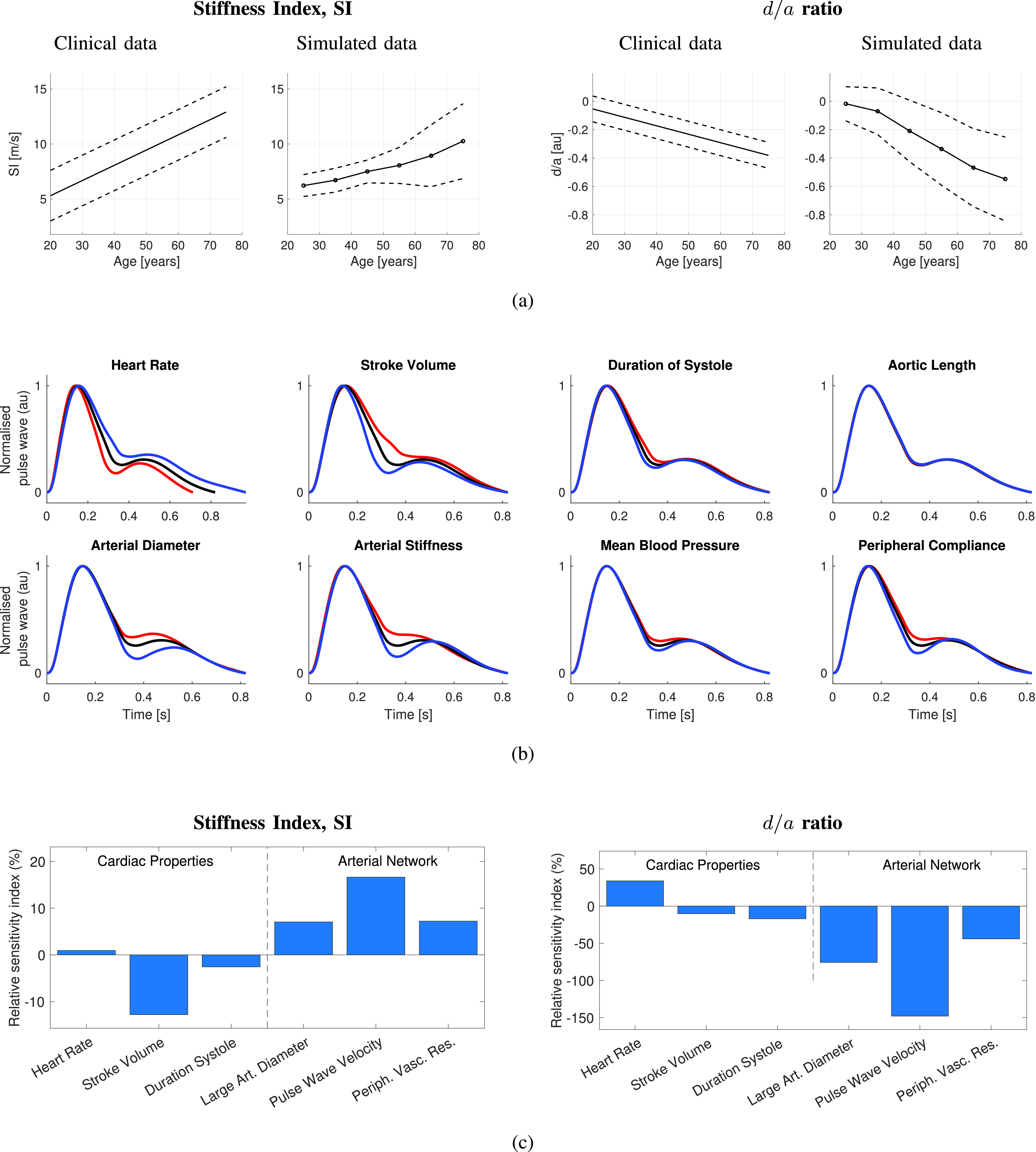
Determinants of PPG pulse wave features: Insights obtained using simulated PPG pulse waves. (a) Comparison of the changes in two pulse wave indices with age observed in simulated and clinical data. (b) Wrist PPG Pulse waves representative of a healthy 25-year old male in black and pulse waves under varying CV properties of ±1 standard deviation from the mean for a healthy individual in red and blue, respectively. (c) Influence of CV properties on two pulse wave features, expressed as the relative sensitivity index: the percentage change in a feature associated with a change in CV parameter of 1 standard deviation from baseline. Sources: data obtained from the PWDB database [114] and analyzed using [265]. Clinical data in (a) obtained from [235].

First, the ability of the model to reproduce changes in a pulse wave shape with age was verified. Fig. 4(a) shows how the SI and $d/a$ changed with age in the simulations. Both indices exhibited similar trends to those observed in humans: the SI increased with age, and $d/a$ decreased with age. This indicates that the modeling accurately captured changes in PPG features with age.

Second, the CV determinants of the pulse wave indices were assessed. In Fig. 4(b), several CV properties were found to have similar effects on the pulse wave: changes in arterial stiffness, mean BP, and peripheral compliance all impacted the portion of the pulse wave between systolic and diastolic peaks; HR and arterial diameter impacted the height of the diastolic peak. The determinants of the SI and $d/a$ are shown in Fig. 4(c). Both indices were predominantly influenced by arterial properties: large artery diameter, PWV, and peripheral vascular resistance. The SI was also strongly influenced by stroke volume, whereas $d/a$ was influenced by HR. These findings are in keeping with clinical observations: the SI and $d/a$ are both influenced by BP (which was altered by changing peripheral vascular resistance in this model) [33], [235], and $d/a$ is influenced by HR [33]. This indicates that the potential utility of the SI and $d/a$ for assessing CV risk may be due at least in part to them being influenced by other CV risk factors, namely, BP and PWV. Additional results relating to a wider range of PPG pulse wave features are presented in the Supplementary Material.

#### 3) Discussion:

Individual features of the pulse wave can be influenced by multiple CV properties. This highlights a potential challenge to using the PPG pulse wave to monitor CV health: algorithms for estimating parameters from the pulse wave must be robust to simultaneous changes in other parameters. For instance, algorithms to estimate BP from the pulse wave must be carefully designed to remain accurate in the presence of changes in arterial stiffness and peripheral compliance, as these properties influenced pulse wave shape similar to BP.

### B. Developing Algorithms to Estimate Physiological Parameters

Algorithms to estimate parameters from the PPG need to perform sufficiently well if they are to be used for clinical use. Algorithms should be able to measure the parameter of interest in the presence of changes in other parameters, despite PPG-derived indices being influenced by multiple parameters. Algorithms may need to combine several pulse wave features and should be tested under a range of CV conditions. Initial development could be performed using data from controlled experiments, including simulated data. Indeed, models have recently been developed to simulate the PPG during bradycardia and ventricular tachycardia [266] and AF [267]. The latter has been used for the initial assessment of an AF detector [268]. Controlled laboratory experiments then allow algorithm performance to be assessed in ideal conditions [45].

Following initial development, algorithms need to be made sufficiently robust to perform well in daily life and any intended clinical settings. Algorithms need to be robust to noise due to poor signal quality and motion artifact. Algorithms may need to be able to identify when parameters cannot be accurately estimated due to insufficient physiological signal content [89]. Algorithm development is aided by datasets of PPG signals containing reference parameters and benchmark algorithms for comparison, as discussed in Section VI.

### C. Acquiring PPG Data During Daily Living

Studies that acquire PPG data during daily living will help identify potential challenges to PPG-based monitoring in this setting and will help develop potential solutions. Potential challenges include the following.

#### 1) Assessing Parameter Repeatability:

A prerequisite to using parameters for clinical decision making is that their measurement should be sufficiently repeatable. Most investigations of the repeatability of PPG-derived parameters have taken place in laboratory settings [269]. Further work should investigate which parameters are sufficiently repeatable when measured in daily life to be used for clinical decision-making.

#### 2) Contextualizing PPG Measurements According to Activities:

The clinical significance of measurements is determined not only by their values (such as an HR > 100 bpm) but also their context (such as whether this measurement was obtained while asleep or shortly after intense exercise). Techniques are required to contextualize measurements according to activities, so they can inform clinical decisions appropriately [123]. For instance, HRs can currently be contextualized according to whether a subject is awake or asleep, and active or inactive (see Section III-D1). If activities, such as stair climbing or intense exercise, could also be identified, then that may provide an opportunity for automated CV risk assessments in daily life (see Section IV-I).

#### 3) Optimizing PPG Measurement Duration and Frequency:

The duration and frequency of PPG-based measurements should be optimized to obtain measurements sufficiently frequently to capture physiological changes (such as recovery from exercise) and infrequent physiological events (such as paroxysmal AF) while prolonging battery life. For instance, the smartwatches in [29] used an adaptive sampling strategy based on physiology and activity. The watch initially attempted to analyze a 1-min PPG signal in a period free from movement every 2 h, and if an abnormality was detected, then this was increased every 15 min [29]. Initial work indicates that PPG signal quality is highest during sleep when movement and ambient light are minimal, supporting the approach of only acquiring PPG signals during periods of low activity [29]. Other approaches to reduce power consumption include: 1) delaying the next measurement after one of low quality [86]; 2) using compressive sampling to reduce the sampling frequency while still being able to accurately obtain information from PPG pulse waves [270], [271]; and 3) using windowed sampling to only sample portions of interest of the PPG pulse wave (such as systolic peaks) [272].

#### 4) Generating Clinical Notifications and Alerts:

Algorithms are needed to generate notifications and alerts from wearable data. In [29], an irregular pulse notification was generated if an irregular pulse was identified in at least five out of six PPG recordings in a 48-h period [29]. Similarly, in [46], algorithms to detect clinical deteriorations in hospital patients generated an alert if at least 50% of the data acquired within a 30-min period were outside of normal ranges. Similar algorithms will be required for each clinical application.

### D. Assessing the Clinical Utility of PPG Monitoring

We now overview the steps required to assess the clinical utility of PPG monitoring during daily living.

#### 1) Validation of Physiological Measurements:

It is important to validate PPG-derived physiological measurements. The first step is to assess algorithm performance. For instance, the performance of algorithms to estimate RR from the PPG has been assessed extensively with studies often assessing the accuracy (or bias) and precision (or variability) of measurements [24]. Ideally, algorithm validation studies would include PPG data from a range of settings (such as different daily activities), acquired using different devices, from different datasets (since performance can differ greatly between datasets [46]). Algorithm performance assessments are useful to device designers for selecting the best algorithm for a device. The second step is to assess the performance of measurements provided by devices [273]. This can inform the choice of device for clinical use and the level of confidence associated with measurements. In both steps, validation studies require precise reference measurements of the parameter of interest, such as reference HRs obtained from simultaneous ECG monitoring [81]. Validation studies provide insight into the clinical applications in which PPG monitoring could be beneficial and allow for an appreciation of the limitations of PPG monitoring. Studies to validate wearable PPG-based devices should be designed to contribute to regulatory approvals required for medical device certification [274] and should follow standard validation protocols where available [170].

#### 2) Suitability for Clinical Decision Support:

Wearable PPG measurements can contribute clinically when they are incorporated into a clinical decision support system to prompt a possible diagnosis, such as a specific disease, or more generally identify a parameter that indicates elevated CV risk.

Research is required to determine whether wearable data can add value to existing risk prediction models. The performance of such systems can be assessed in two steps. First, the system’s performance for classifying patients according to whether they have the disease (or risk factor) or not should be assessed. A system’s classification performance can be assessed by its sensitivity and specificity [193]. At this stage, the system can be assessed in a convenient sample of patients (for instance, equal numbers of controls and patients with the disease). Second, its real-world performance should be assessed in the target population using statistics such as positive and negative predictive values, which takes into account disease prevalence. This is important as diagnoses, such as AF, are present in a small minority of the population, so even a system with high sensitivity and specificity (e.g., 95% for each) can result in relatively low PPV (50% in this example) when used in a population with low disease prevalence (e.g., 5%): further details of these calculations are provided in the Supplementary Material. A low PPV indicates a high frequency of false alerts, at best increasing healthcare resource utilization, and at worst resulting in misinformed clinical decisions. Since a system’s clinical utility is determined not only by its classification performance but also by disease prevalence, it should be evaluated in the context of the healthcare pathway for which it is intended.

The use of wearables for clinical decision support must be not only effective but “do more good than harm at a reasonable cost” [275]. First, the benefits of using wearables must outweigh the costs. It is important to consider the costs of: 1) providing devices to patients who do not have their own device; 2) training patients to wear and use the device; 3) clinical review of data; and 4) subsequent clinical assessment and treatment resulting from any new findings. Second, there is a need to understand the potential harms caused by using wearables and minimize them. For instance, false positives issued in a screening program may cause distress, and the act of being screened may cause anxiety.

### E. Integration Into Clinical Pathways

The final step to realizing the full potential of wearable PPG-based monitoring is to integrate it into clinical pathways. We now describe five possible models for integration and summarize the key requirements for each model.

#### 1) Screening Programs:

PPG-based wearables may have a role to play in screening programs for diseases such as AF [276], [277]. The performance requirements depend on the screening program design: a PPG-based device could be used as an initial screening tool, prompting further testing in those subjects exhibiting possible AF, or potentially, it could be used as the sole device in a screening program. Currently, a diagnosis of AF requires ECG verification, so the potential role of PPG-based devices is limited to initial assessment. In this context, a PPG-based system requires high sensitivity and moderate PPV so that most patients with AF are identified, and the workload associated with further testing is manageable. In contrast, PPG-based devices would need both high sensitivity and high PPV in order to be considered as potential alternatives to current ECG-based screening strategies. This is particularly important in the case of AF because subsequent anticoagulant treatment has the side effect of increased bleeding risk.

#### 2) Patient-Led Measurements to Prompt Clinical Assessment:

PPG-based wearables could also be used by patients to prompt clinical assessment. For instance, a patient’s own device may notify them of a possible arrhythmia. On reporting this to a physician, the physician may conduct further investigation in order to confirm or deny the diagnosis, such as ECG-based monitoring. For this model, PPG-based systems require moderate PPV to reduce unnecessary healthcare resource utilization. There are currently significant issues with the widespread use of consumer devices in this manner. First, even the best devices may not perform sufficiently well to avoid excessive resource utilization. Second, a wide range of consumer devices is available with varying performance levels and no universal standards. Third, this model is limited to patients who can afford to use, and choose to use, a wearable device. Fourth, further research is required to determine whether device-detected asymptomatic disease confers the same risk as that detected in clinical practice and whether it should be treated in the same manner [276]. Nonetheless, patient-led device use could confer substantial patient benefit in the future as the performance and capability of devices improve.

#### 3) Supplementary Clinical Use in Specific Settings:

PPG-based devices could provide supplementary continuous monitoring in settings where monitoring is, otherwise, limited to intermittent measurements (such as acute hospital wards). This could reduce delays in detecting physiological changes, which may prompt further investigation and treatment. The key requirement is the high accuracy of PPG-derived parameters, allowing changes in parameters to be tracked. Sufficient accuracy minimizes the frequency of false alerts, reducing excess demands on healthcare staff. This model often supplements routine practice, rather than replacing it, whereby PPG-derived measurements are only used to prompt additional assessments rather than to make treatment decisions.

#### 4) Self-Directed CV Health Monitoring:

PPG-based devices may have utility for guiding the self-management of CV health. For instance, device measurements relating to fitness and mental stress could prompt users to change their lifestyle by prompting additional exercise or minimizing exposure to stressful situations. In this model, the consequences of erroneous readings would be less severe, as users would only be prompted to undertake beneficial lifestyle changes. Therefore, device requirements are less stringent. At present, there is little evidence for the long-term health benefits of this approach. Future research should investigate whether it can effectively modify CV risk factors (such as BP or HRR after exercise) and then whether they have long-term benefits.

#### 5) Population-Level Infectious Disease Monitoring:

The use of wearables to track population-level physiological changes associated with infectious diseases does not require as accurate parameter estimates as other models for two reasons. First, this model could analyze temporal changes in parameters rather than absolute values, reducing the need for accurate parameter estimation (although still requiring sufficient precision). Second, this model uses data from many individuals to track geographical trends, limiting the impact of individual errors.

## VI. Research Resources

This section presents resources for conducting research into wearable photoplethysmography: PPG datasets, PPG analysis tools, and wearable PPG devices.

### A. PPG Datasets

Table 2 presents a summary of datasets containing PPG signals suitable for research. Several datasets were acquired during routine clinical practice (e.g., MIMIC, CapnoBase, and University of Queensland datasets), where as others were recorded from volunteers. The table provides details of the additional signals available in the datasets and the recording conditions, indicating the types of research questions that could be addressed using each 2 dataset. Many datasets contain finger PPG signals, whereas only a few contain wrist PPG signals: pulse wave morphology may differ between these sites [58]. Furthermore, very few datasets have been acquired in daily life conditions (the PPG-DaLiA Dataset is a notable exception [295]), making it difficult to compare wearable PPG analysis techniques [28], [72].

**TABLE 2:** Datasets of PPG Signals. Definitions: Resp—Respiratory Signal; PCG—Phonocardiogram; Accel—Acceleromertry; Gyro—Gyroscope; EDA—Electrodermal Activity; EMG—Electromyogram; GSR—Galvanic Skin Response; and ICP—Intracranial Pressure

Dataset	Ref	No subjs	Other signals	Description
U.K. Biobank (Field 4205)	–	205,357	–	Single finger PPG waves from middle-aged subjects.
MIMIC Critical Care Database	[278]	10,000s (growing)	ECG, BP, resp, others	Recordings from critically-ill adults and neonates, lasting from minutes to days. Typically at finger.
VitalDB	[279]	6,153	ECG, BP, resp, others	Finger PPG recordings from patients during operations.
MESA Dataset	[280]	2,056	ECG, resp, others	Finger PPG recordings from adults undergoing polysomnography.
SOMNIA Database	[281]	100s (growing)	ECG, resp, others	Wrist PPG recordings from children and adults undergoing polysomnography.
PPG-BP Database	[282]	219	–	Three finger recordings from adults aged 20–89 with and without CVD, ≈ 3 waves per recording.
Sleep Disordered Breathing Database	[283]	146	–	Finger recordings lasting ≥3 hours, acquired from children referred for polysomnography.
ECSMP	[284]	89	ECG, accel, others	Wrist recordings acquired from healthy subjects during a protocol designed to induce different emotions.
Pulse transit time during vasoconstriction Dataset	[285]	86	ECG, BP	≈ 35-min wrist and finger recordings during cold pressor and active stand tests.
Vortal Dataset	[45] [73]	57	ECG, resp	10-min finger and ear recordings before and after exercise from healthy adults aged 18–39 and > 70.
BIDMC	[286]	53	ECG, BP, resp	8-min recordings from critically-ill adults (a subset of the MIMIC-II dataset).
CapnoBase	[287]	42	ECG, resp	8-min recordings from pediatrics and adults during elective surgery and anesthesia.
Bed-based BCG Dataset	[288]	40	ECG, BCG, BP	Recordings from adults whilst at rest.
DEAP Database	[289]	32	ECG, resp, video, others	Thumb recordings from young, healthy subjects whilst watching one-minute 40 videos.
University of Queensland Vital Signs Dataset	[290]	32	ECG, resp, BP, EEG	Recordings from patients during anesthesia, ranging from minutes to hours in duration.
ESUM SNF Project Dataset	[291]	31	accel, EDA	Wrist recordings whilst walking.
MARSH Dataset	[292]	29	ECG, resp	15-min finger recordings during spontaneous and metronome-guided breathing.
Noninvasive BP Estimation	[150]	26	ECG, BP, PCG	Finger recordings from healthy adults.
gyro-acc-ppg Dataset	[293]	24	ECG, accel, gyro	Wrist recordings from healthy subjects during an exercise protocol lasting 12 minutes.
Wearable and Clinical Devices Dataset	[294]	18	ECG, resp, accel, EDA	Wrist PPG recordings acquired for 5 mins at rest, and 5mins whilst walking on spot.
PPG-DaLiA Dataset	[295]	15	ECG, resp, accel, EDA	Recordings acquired for ≈ 2.5 hours during a protocol of daily living activities.
WESAD Dataset	[296]	15	ECG, resp, accel, others	Recordings acquired in a ≈ 2 hour protocol designed to amuse, stress, and relax.
iAMwell Dataset	–	15	ECG, resp	≈ 20-min recordings before, during and after running.
Simultaneous Measurements Dataset	[297]	13	ECG, accel, resp	Recordings from adults at rest and during cognitive and physical tasks.
IEEE Signal Processing Cup 2015	[81]	12	ECG, accel, two PPGs	≈ 5-min recordings during intensive physical exercise from males aged 18–35.
AffectiveROAD Dataset	[298]	10	accel, EDA	Recordings acquired whilst driving a car for ≈ 86 minutes from mostly young adults.
Labeled raw PPG Signals	[299]	9	–	Finger recordings acquired at rest for ≈ 20−40 minutes from healthy adults.
Wrist PPG During Exercise	[300]	8	ECG, gyro, accel	Wrist recordings acquired from adults aged 22–32 during walking, running and bike riding.
PPG-ACC Dataset	[301]	7	accel	Wrist recordings acquired from healthy adults aged 20–52 during rest, squatting and stepping.
Raw PPG Signal in Varying Levels of Activity	[240]	5	–	Finger recordings acquired at rest, talking and walking for ≈ 10 mins each from healthy adults.
PPG-Diary	[72]	1	two PPGs	A 28-day thumb recording from a healthy adult, with annotations of activities of daily living.
Eight-Emotion Sentics Dataset	[302]	1	EMG, GSR, resp	25-min recordings during a protocol to evoke emotions from 1 subject each day for 20 days.
Pulse Wave DataBase	[114]	4,374 (synthetic)	BP, blood flow, others	Single simulated PPG pulse waves representative of healthy adults aged 25–75.
Photoplethysmography in dogs & cats	[303]	21 (animals)	three PPGs	≈ 10−20 sec recordings at several arterial sites from 11 dogs and 10 cats.

### B. PPG Analysis Tools

Several tools have been created to analyze beat-to-beat interval data in order to assess HRV (summarized in [308, Table 1]), including a benchmarked toolbox to extract and analyze IBIs from PPG, ECG, and BP signals [308]. *PulseAnalyse* is a MATLAB tool for analyzing PPG and BP pulse waves [114], [309]. It performs several steps: 1) beat detection; 2) signal quality assessment; 3) filtering; 4) calculating an average pulse wave; 5) calculating pulse wave derivatives; 6) identifying fiducial points; and 7) calculating pulse wave indices. This allows the nonexpert to analyze PPG signals for research.

### C. Wearable Devices for Acquiring PPG Signals

Many wearables are equipped with a PPG sensor (see [310, Table 1]). However, most do not provide access to the PPG signal, limiting their utility in research. Table 3 lists a selection of wearable devices that can be used to acquire and record PPG data. The performance of these devices varies and can have a considerable impact on the success of a study, influencing the proportion of time for which data are captured, whether data capture is biased toward particular subjects, and the quality of data in different activities [72].

**TABLE 3:** Selection of Devices That Have Been Used to Acquire PPG Signals Continuously From Ambulatory Subjects. Definitions: GSR—Galvanic Skin Response; Temp—Temperature; Accel—Accelerometry; and BT—Bluetooth

Device	Ref	Other Signals	Site	Data capture
Biostrap Biometric Set	[304]	two PPGs, accel, SpO_2_	Wrist	BT, storage
Biovotion Everion	[305]	accel, GSR, SpO_2_	Arm	BT
Cossinus° One / Two	[306]	accel, temp, SpO_2_	Ear	BT
Empatica E4 Wristband	[307]	GSR, temp, accel	Wrist	BT, storage
Nonin 3150 WristOx_2_	[86]	SpO_2_	Finger	BT
Polar OH1	[65]	accel	Arm (flexible)	BT
PulseOn OHR Tracker	[194]	accel	Wrist	BT, storage
SmartCare pulse oximeter	[72]	two PPGs, SpO_2_	Finger	BT

## VII. Conclusion

This review has demonstrated the exciting opportunity afforded by wearable PPG devices to monitor CV health in daily life and to potentially provide rich information to aid clinical decision-making in the future. Key conclusions arising from the review are given as follows.

1) Even though photoplethysmography has been used clinically for 40 years in pulse oximeters, there is still much opportunity to exploit the technology for further benefit, aided by the widespread use of photoplethysmography in wearable devices.
2) The PPG signal contains a wealth of information on the CV system although there are several confounders that can either obscure this information (such as motion artifact) or alter it (such as contact pressure).
3) Key signal processing challenges include the estimation of BP, RR, and arterial blood oxygen saturation (using reflectance photoplethysmography), each of which could be of great value clinically.
4) Several promising clinical applications for wearable PPG devices have been identified. The detection of an irregular pulse to prompt further assessment for AF is, perhaps, nearest to being ready for clinical use.
5) Care must be taken to ensure that wearable PPG devices meet the standards required for clinical use. The data provided by devices must be both accurate and useful. This can be achieved by using quality assessment to only output parameters when they are accurate and using other sensors, such as accelerometry, to identify periods of standardized activities (such as while resting or asleep).
6) There are several approaches to integrate wearable PPG-based devices into clinical pathways, each of which has different performance requirements.
7) Further development of the technology will be aided by freely available datasets, particularly those acquired in daily living with reference labels, and open-source algorithms against which to compare new signal processing techniques.
