# Supplementary material for: Wearable Photoplethysmography for Cardiovascular Monitoring
Source: Proc IEEE Inst Electr Electron Eng. Author manuscript; Available in PMC 2022 Mar 29. (PMC7612541; doi:10.1109/JPROC.2022.3149785)
Supplement: Supplementary file 1 [file EMS143850-supplement-Supplementary_Material.pdf]

Supplementary Material accompanying:

Wearable photoplethysmography for cardiovascular monitoring

Peter H. Charlton, Panicos A. Kyriacou, Jonathan Mant, Vaidotas Marozas, Phil Chowienczyk, and Jordi Alastruey

This supplementary material should be read alongside the article, as several references are made to the main text.

I. Additional Images

A. Wrist cross section

Fig. 1 (a) in the main text shows a cross section of the wrist with a PPG sensor positioned on the surface. Fig. SM1 shows a similar cross section of the wrist without a PPG sensor.

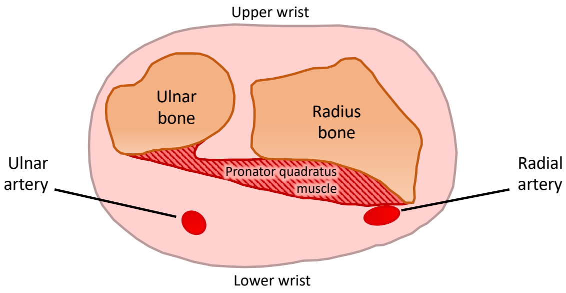

Fig. SM1. A cross section of the wrist.

Source: adapted from Fig. 421, titled 'Transverse section across distal ends of radius and ulna', in [SM1] (public domain).

B. Finger cross section

Fig. 1 (c) shows a cross section of the finger with a PPG sensor positioned on the surface. A complementary image showing a similar cross section of the finger is provided in Fig. SM2. Dorsal digital arteries in the upper finger are not shown based on the assumption that their contribution to the PPG signal is relatively minor (given that they terminate at approximately the mid-proximal phalanx [SM2]).

C. Applanation tonometry at the wrist

Some smart wearables can also measure an uncalibrated BP signal at the wrist using applanation tonometry [SM3], as shown in Fig. SM3. In applanation tonometry a plunger is used to flatten the wall of the radial artery

Please see the main article for author affiliations and the acknowledgment.

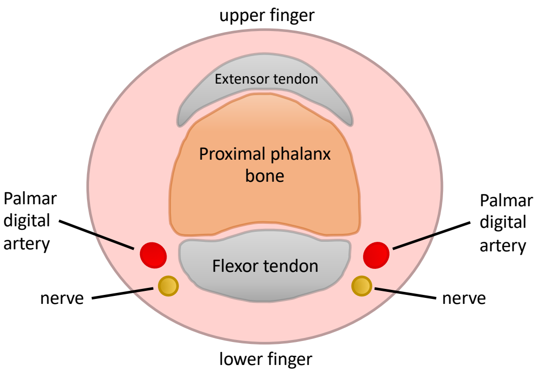

Fig. SM2. A cross section of the finger, at the proximal phalanx.

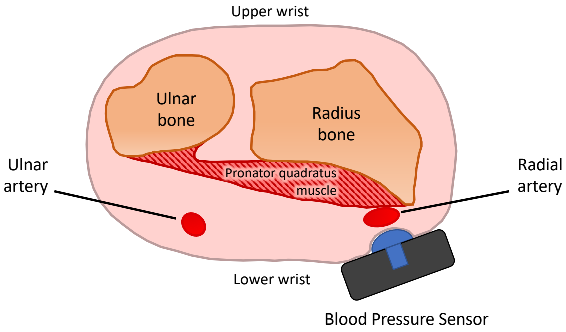

Fig. SM3. Using applanation tonometry to measure blood pressure pulse wave signals at the wrist.

Source: adapted from Fig. 421, titled 'Transverse section across distal ends of radius and ulna', in [SM1] (public domain).

so that the pressure on the plunger can be assumed to be uniform across its surface, and equal to the intra-arterial pressure. The plunger needs to be carefully placed over the radial artery, and is prone to becoming dislodged [SM4]. Fig. SM4 shows both an applanation tonometry sensor and a PPG sensor.

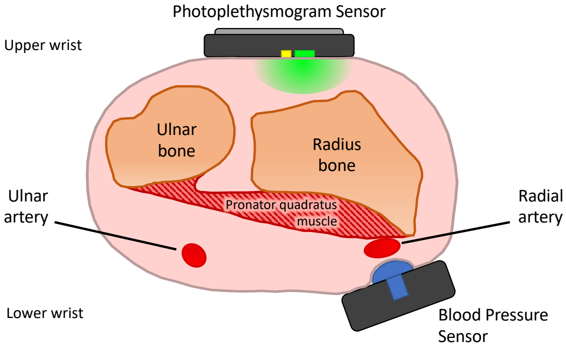

Fig. SM4. Using photoplethysmography and applanation tonometry to measure arterial pulse wave signals at the wrist.

Source: adapted from Fig. 421, titled 'Transverse section across distal ends of radius and ulna', in [SM1] (public domain).

D. Origins of the PPG

Fig. 1 (d) shows the simplified origins of light attenuation which form the PPG signal (arterial blood, venous blood and other tissues). An additional image is provided for educational purposes in Fig. SM5 - this can be used to introduce the PPG signal before describing its origins.

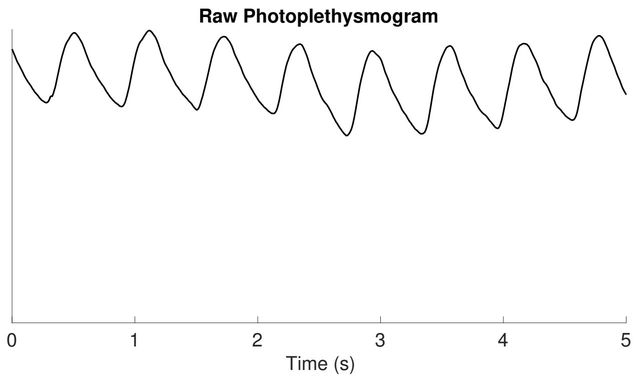

Fig. SM5. An additional image relating to the origins of the PPG signal.

Source: adapted from [SM5] under CC BY 4.0. The data in the images are modified from original data taken from the MIMIC Waveform Database [SM6].

E. Influence of breathing on the PPG

In Section II. B. (2) the influence of breathing on the PPG signal was summarised. The three principal effects of breathing on the PPG are illustrated in Fig. SM6.

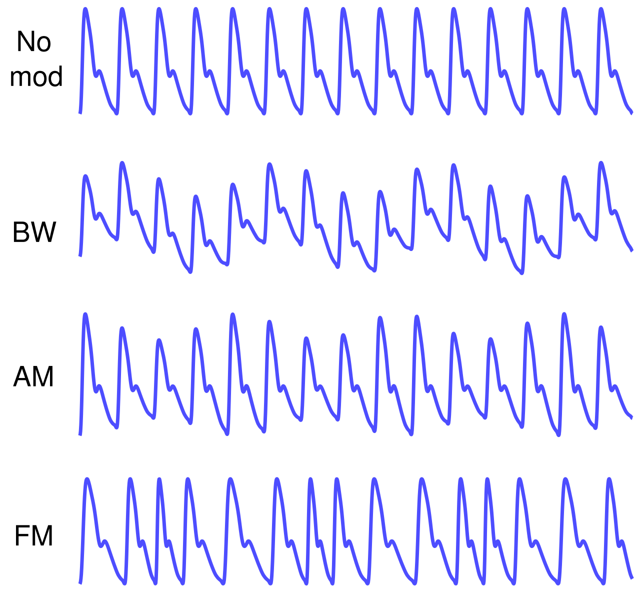

Fig. SM6. The effects of breathing on the PPG, showing three idealised respiratory modulations: baseline wander (BW), amplitude modulation (AM), and frequency modulation (FM).

Source: adapted from [SM7] under CC BY-NC, and [SM8] under CC BY 4.0.

II. Determinants of PPG pulse waves: Analysis using computational modelling

The results of an *in silico* study of the determinants of two PPG pulse wave features are presented in Fig. 4. Additional results relating to a wider range of PPG pulse wave features are presented in Figs. SM7 and SM8.

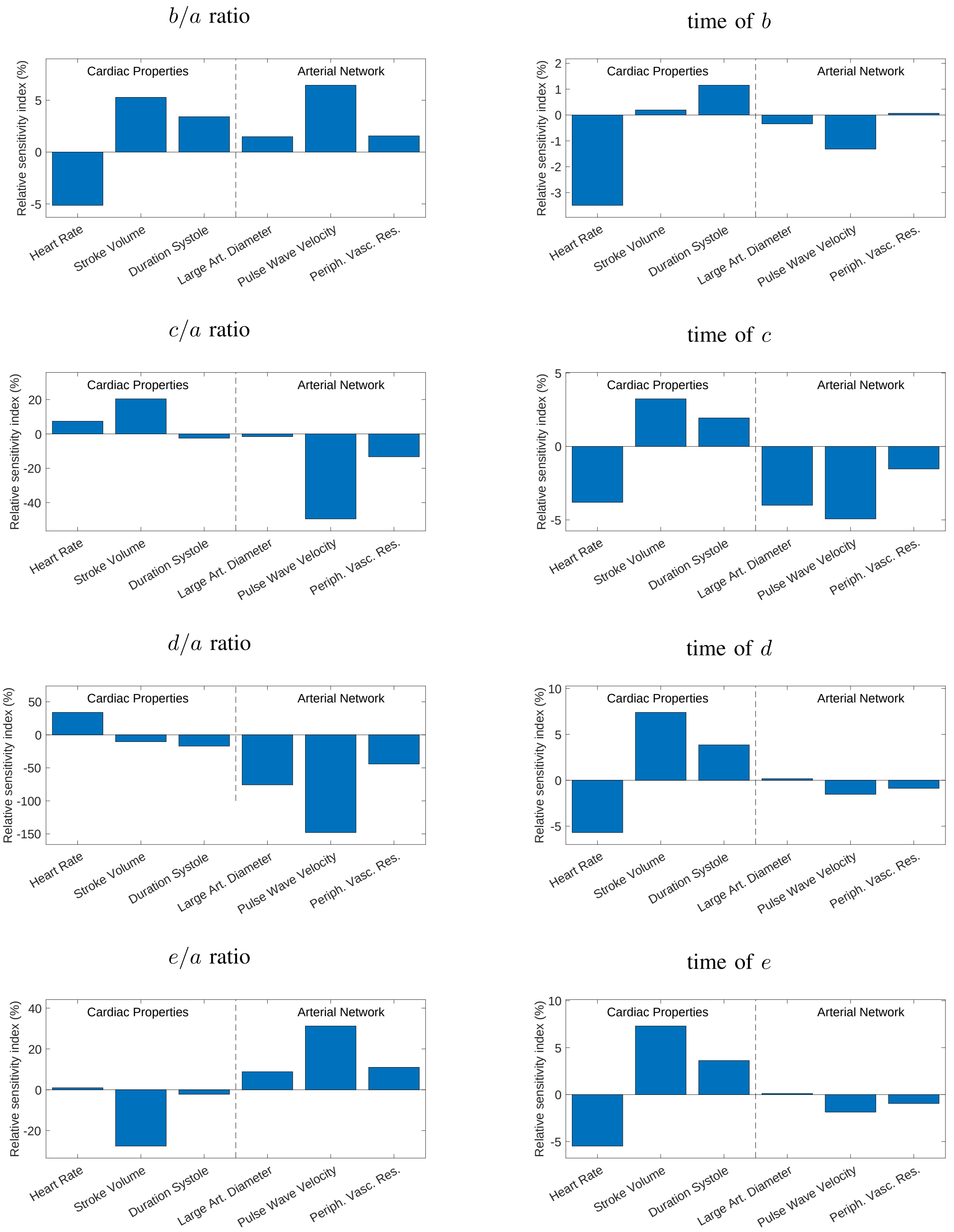

Fig. SM7. The influence of CV properties on PPG features derived from the second derivative.

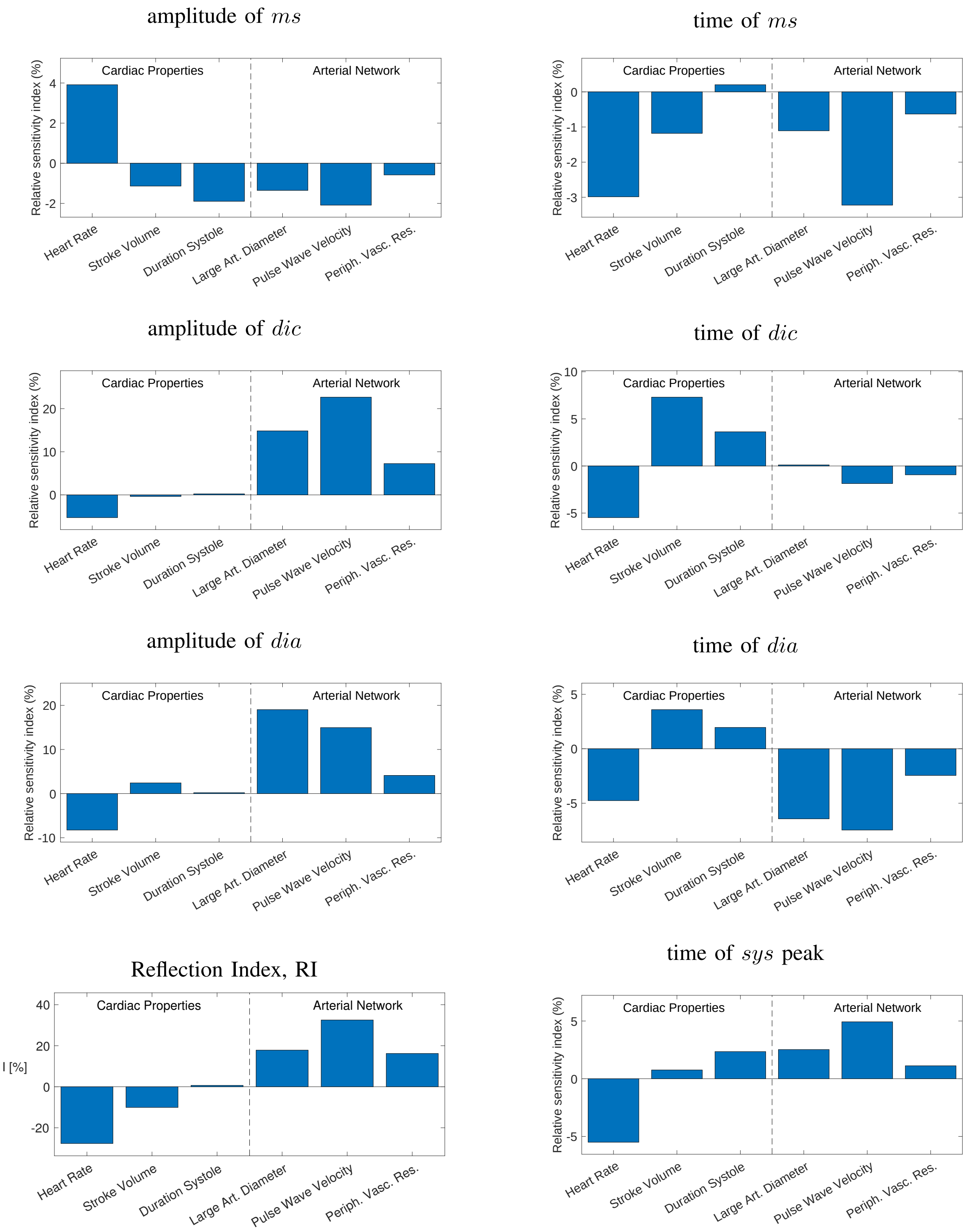

Fig. SM8. The influence of CV properties on PPG features (continued from Fig. SM7).

III. The importance of assessing real-world clinical utility of diagnostic tests

Section V. D. (2) demonstrated the importance of assessing the real-world clinical utility of PPG-based diagnostic tests. We used the example of a system for detecting AF, which could result in a relatively low positive predictive value (PPV) despite a high sensitivity and specificity because of a low disease prevalence in the target population. We now provide details of the calculations used.

Example system's classification performance:

$$\text{sensitivity, } sens = \frac{TP}{P} = 95\% = 0.95$$

$$\text{specificity, } spec = \frac{TN}{N} = 95\% = 0.95$$

where

$$\text{number of true positives} = TP$$

$$\text{number of true negatives} = TN$$

$$\text{number of positives in sample} = P$$

$$\text{number of negatives in sample} = N$$

Consider assessing the system's real-world performance, such as in a sample of elderly community dwelling subjects with a disease prevalence of 5%, using the positive predictive value (PPV) which is insightful when considering whether to use this system in a screening programme:

$$\text{number of subjects} = n$$

$$\text{disease prevalence} = p = 0.05$$

$$\text{number of positives in sample,} \quad P = np$$

$$\text{number of negatives in sample,} \quad N = n(1-p)$$

Positive predictive value,

$$PPV = \frac{sens \times P}{sens \times P + (1 - spec) \times N}$$

$$PPV = \frac{sens \times np}{sens \times np + (1 - spec) \times n(1-p)}$$

$$PPV = \frac{0.95 \times 0.05}{0.95 \times 0.05 + (1 - 0.95) \times (1 - 0.05)} = 0.5 = 50\%$$

References

[SM1] H. Gray, *Anatomy of the human body*, american 2 ed., W. H. Lewis, Ed. Philadelphia and New York: Lea and Febiger, 1918.

[SM2] T. Endo, T. Kojima, and Y. Hirase, "Vascular anatomy of the finger dorsum and a new idea for coverage of the finger pulp defect that restores sensation," *Journal of Hand Surgery*, vol. 17, no. 5, pp. 927–932, 1992. https://doi.org/10.1016/0363-5023(92)90472-2

[SM3] N. Kizilova, "Review of emerging methods and techniques for arterial pressure and flow waves acquisition and analyses," *International Journal of Biosensors and Bioelectronics*, vol. 4, no. 4, pp. 179–187, 2018. https://doi.org/10.15406/ijbsbe.2018.04.00122

[SM4] T. Komori *et al.*, "Comparison of wrist-type and arm-type 24-h blood pressure monitoring devices for ambulatory use," *Blood Pressure Monitoring*, vol. 18, no. 1, pp. 57–62, 2013. https://doi.org/10.1097/MBP.0b013e32835d124f

[SM5] P. H. Charlton, "Capitalising on smart wearables to improve health monitoring," Zenodo: http://doi.org/10.5281/zenodo.1406011, 2018. http://doi.org/10.5281/zenodo.1406011

[SM6] A. E. Johnson *et al.*, "MIMIC-III, a freely accessible critical care database," *Scientific Data*, vol. 3, p. 160035, 2016. https://doi.org/10.1038/sdata.2016.35

[SM7] P. S. Addison *et al.*, "Developing an algorithm for pulse oximetry derived respiratory rate (RR(oxi)): a healthy volunteer study," *Journal of Clinical Monitoring and Computing*, vol. 26, no. 1, pp. 45–51, 2012. https://doi.org/10.1007/s10877-011-9332-y

[SM8] P. H. Charlton *et al.*, "An assessment of algorithms to estimate respiratory rate from the electrocardiogram and photoplethysmogram," *Physiological Measurement*, vol. 37, no. 4, pp. 610–26, 2016. [Online]. Available: https://doi.org/10.1088/0967-3334/37/4/610
